# EHMTI-0155. The course of headache in idiopathic intracranial hypertension: a 12 month prospective follow-up study

**DOI:** 10.1186/1129-2377-15-S1-C64

**Published:** 2014-09-18

**Authors:** H Yri, C Rönnbäck, M Wegener, S Hamann, R Jensen

**Affiliations:** 1 Danish Headache Center, Neurology, Copenhagen, Denmark; 2 Ophthalmology, Opthalmology, Copenhagen, Denmark; 3 Neuro-ophthalmology, Opthalmology, Copenhagen, Denmark; 4 Neuro-ophtalmology, Opthalmology, Copenhagen, Denmark

## Background

Although many patients with Idiopathic intracranial hypertension (IIH) experience disabling headache, data on the long-term outcome are sparse. Thus very little is known about predictors and mechanisms of chronification.

## Aims

To prospectively describe the course of headache during the first year of IIH.

## Methods

Patients with newly diagnosed IIH were consecutively included from December 2010 to June 2013. Treatment according to international guidelines was initiated at diagnosis. Headache history was based on diaries and standardized interviews performed at baseline and after 1, 2, 3 and 12 months. Intracranial pressure (ICP) was recorded at base line and after 3 months. Papilledema was assessed by optical coherence tomography (OCT) and compared to healthy controls.

## Results

We included 44 patients, 35 completed the one-year follow-up. Dramatic headache improvement occurred within the first weeks after diagnosis and treatment. After one year 13 patients reported no headache. Fifteen of the remaining 22 patients reported sustained chronic headache. Episodic headache was reported by 7 patients. Early age of onset (OR=1.13, p=0.03) and high ICP (and OR=0.86, p=0.03) were associated with positive headache outcome. Papilledema decreased rapidly within the first three months of diagnosis. After 12 months OCT measures had normalised.

## Conclusions

Although headache responded very well in 43% of patients, sustained long-term headache was seen in the remaining patients, despite normalization of ICP and papilledema. Headache in IIH may thus be attributed to more complex mechanisms than ICP elevation alone. High ICP at baseline and young age predicted positive headache outcome.

Conflict of interest.

